# 

**Published:** 1856-10

**Authors:** 


☞ Dr. Goadby, of the "*Medical Independent*" of Detroit, Michigan, has prosecuted Dr. Zina Pitzer, President of the American Medical Association, for slander. We have not learned what were the offensive words spoken.

☞ We are in receipt of a number of new and valuable works, which will be noticed in our next number.

